# A Short-Term Effect of Wearable Technology-Based Lifestyle Intervention on Body Composition in Stage I–III Postoperative Breast Cancer Survivors

**DOI:** 10.3389/fonc.2020.563566

**Published:** 2020-10-20

**Authors:** Changming Zhou, Miao Mo, Zezhou Wang, Jie Shen, Jiajian Chen, Lichen Tang, Jiajia Qiu, Yiqun Ling, Huiping Ding, Qin Jiang, Hui Wang, Zhimin Shao, Ying Zheng

**Affiliations:** ^1^Department of Cancer Prevention, Fudan University Shanghai Cancer Center, Shanghai, China; ^2^Department of Oncology, Shanghai Medical College, Fudan University, Shanghai, China; ^3^Department of Breast Surgery, Fudan University Shanghai Cancer Center, Shanghai, China; ^4^Department of Nutrition, Fudan University Shanghai Cancer Center, Shanghai, China; ^5^Shanghai Ruochu Information Technology Co., Ltd., Shanghai, China; ^6^Huami Information Technology Co., Ltd., Beijing, China

**Keywords:** breast cancer, body weight, body composition, life-style intervention, wearable technology

## Abstract

**Background and Aim:**

A healthy body composition can improve the prognosis of breast cancer survivors. The study aimed to describe the body composition profile of breast cancer survivors and find out whether a short-term (3 months) wearable device-based lifestyle intervention had an effect on patients’ body weight and body composition.

**Methods:**

A before-and-after study was conducted on patients with stage I–III postoperative breast cancer, aged 18–70 years. Body composition was analyzed at baseline, and then patients went for a health education program. A wearable activity tracker and a goal of calorie consumption based on each individual’s weight were provided to each participant, and they were required to be equipped for 90 days. After 3 months, body composition was analyzed again.

**Results:**

Of 113 patients who completed the study, 65.49% showed a normal body mass index (BMI) at baseline assessment, 71.68% had a body fat percentage of more than 30%, and 41.59% had less skeleton muscle mass. During the intervention, the daily step count was 8,851.28 ± 2,399.31, and 59.21% reached the set goal calorie consumption. After a 3-month intervention, the patients had a significant reduction in body weight, fat mass, BMI, body fat percentage, and visceral fat area, but not in protein mass and skeleton muscle mass. Patients of different age, molecular classification, and therapy benefited from the intervention.

**Conclusion:**

Wearable technology with body composition analysis and health education for breast cancer survivors may help reduce weight and improve body composition even in a short time.

**Clinical Trial Registration:**

http://www.chictr.org.cn/showproj.aspx?proj=40672, identifier ChiCTR1900024258.

## Background

Breast cancer is the most common cancer among women, with more than 2 million new cases worldwide each year (1). The survival of patients with breast cancer has greatly improved with enhanced screening programs and advance treatment strategies. The 5-year survival rate of breast cancer in China is 83.2% (2) and more than 90% in developed regions such as Shanghai (3). The prognosis of patients with breast cancer needs to be improved. It is hard to evaluate the effect of the intervention on survival through a short-term trial because the survival of breast cancer is relatively good, and events are hard to be observed during a short term.

A previous study showed that patients with breast cancer had an increase in body weight of 2.7 kg during chemotherapy (4). The reason for the body weight gain during chemotherapy could be complex, including overeating related to psychological factors, decreased physical activity, use of steroids, and loss of ovarian function associated with chemotherapy, which results in hormonally mediated changes in fat accumulation and distribution (5). Increasing evidence showed that weight was associated with the risk of breast cancer, especially ER+/PR+ invasive breast cancer, among postmenopausal women (6), and high body weight and high body fat were related to poorer outcomes, with an increased mortality risk of 17% per 5 kg/m^2^ (7). The summary relative risk of total mortality was 1.41 and 1.07 for obese and overweight women, respectively, compared with normal-weight women (8). Evidence also showed that high body fat increased the risk of all-cause mortality and breast cancer mortality during both pre-diagnosis and post-diagnosis periods (9). The obese adipose tissue can release or regulate more than 50 different cytokines, chemokines, and hormone-like factors, including HIF-1, leptin, and adiponectin (9) (10), which promote angiogenesis by turning on vascular endothelial growth factor. The released free fatty acids can also activate the NF-κB pathway, which increases aromatase expression and estrogen synthesis (11). Hence, body weight, body mass index (BMI), body fat, or physical activity status may serve as intermediary indicators predicting the survival of breast cancer survivors.

Good results were achieved in patients’ weight control. Studies showed that physical activities reduced the recurrence, breast cancer-specific mortality, and all-cause mortality (12–15). However, these studies mostly focused on obese patients, and the intervention was intensive monitoring through large workloads such as telephone supervision (16), which made it hard to be popularized.

Wearable technology, such as wearable activity trackers, has the potential to facilitate these activities through continuous monitoring and feedback (17), even with health promotion and peer support; each device is less expensive. This technology has been widely used in the management of diabetes, physical inactivity, and smoking (18). It also can potentially benefit cancer survivors who have a comparatively good prognosis, such as in breast cancer and in colorectal cancer. However, studies using such devices and technology are limited because this technology has bloomed only in recent years.

This study aimed to describe the body composition profile of general breast cancer survivors and find out whether a short-term (3 months) wearable device-based lifestyle intervention had an effect on patients’ body composition, such as fat mass, body fat percentage, and skeleton muscles, and to explore the technology’s acceptance among breast cancer survivors.

## Materials and Methods

### Study Design

A before-and-after comparison was conducted among the eligible patients in Fudan University Shanghai Cancer Center. Data on demographics, dietary status, quality of life (QoL), and body composition were collected at baseline. Then, a wearable technology-based lifestyle intervention, which facilitated both data collection and physical activity supervision, was offered to the participants. After 3 months, the patients were required to fill in the QoL questionnaire and undergo a test on body composition again, with satisfaction and adverse effects recorded.

### Patients

Patients with stage I–III postoperative breast cancer, aged 18–70 years, were included in the program. The inclusion criteria were as follows: (1) all patients needed to complete major treatment, including surgery, adjuvant chemotherapy, or adjuvant radiotherapy, for at least 1 year but could be under endocrine therapy and (2) all patients were required to be equipped with a smartphone for app installation. The exclusion criteria were as follows: (1) with distant metastasis, (2) pregnant or breastfeeding, (3) with a severe mental disorder or substance abuse, (4) with diseases or injuries, making them unable to take part in physical activities, including fracture, limb disability, and rheumatic arthritis, and (5) those who already joined other physical activity-related programs. Patients who applied for the program were screened using an online questionnaire. The participants were scheduled to be selected from all the eligible patients using a simple random sampling method.

### Intervention

A wearable activity tracker (Mi band 2, developed by Huami Corporation and based on the Amazfit health cloud data services) was given to the participants, and they were guided to use the equipment. Then, face-to-face health education, as well as a pamphlet on lifestyle guidelines for breast cancer survivors, was provided to each participant according to the guidelines on lifestyle modification for Chinese breast cancer survivors (19), emphasizing on at least 150 min of moderate-to-vigorous physical activities (MVPAs) per week. An equivalent of 30-min moderate physical activity calorie consumption goal was set based on each participant’s weight. The participants were suggested to complete the calorie consumption goal daily during the 3-month intervention. They could get access to the real-time calorie consumption data from the app (Mi Fit) synchronized with the equipment.

### Data Collection

The demographic information of patients was collected using a structured questionnaire. Data on dietary status were collected using a food frequency questionnaire. Both parts were conducted online and filled in under the guidance of a trained investigator. The QoL was assessed using the Functional Assessment of Cancer Therapy—Breast Cancer scale developed by Cella (20) and translated into simplified Chinese. It comprised 27 general items in four domains (physical well-being, social/family well-being, emotional well-being, and functional well-being) and nine items in breast cancer-specific domain (21). Disease- and treatment-related information was collected from a structured breast cancer database. Body weight, protein mass, fat mass, skeletal muscle mass (SMM), BMI, percentage body fat (PBF), basal metabolic rate (BMR), and visceral fat area (VFA) were measured as body composition indicators by the bioelectrical impedance analysis method (22) using an InBody S10 body composition analyzer. The activity data collection included step numbers, walking time, running time, daily calorie consumption (based on the activities monitored), daily running-specific calorie consumption, and sleep status (awake, deep sleep, and light sleep). The data were stored in the band for up to 30 days and, once linked to the app (Mi Fit), they were synchronized to the user’s account. With prior authorization from all the participating patients, the daily statistics data of each patient were transferred to a patients’ health management system developed by Shanghai Ruochu Information Technology Co., Ltd.

### Statistical Analysis

Number and percentages were used for describing categorical variables, while means ± standard deviation and median (quartiles) were used for describing measurement data depending on whether the distribution was normal. Paired-sample *t* test was used to analyze the before-and-after body composition at a statistically significant level (α = 0.05). All analyses were performed using SPSS 21.0 (IBM Corp., United States).

## Results

**FIGURE 1:**
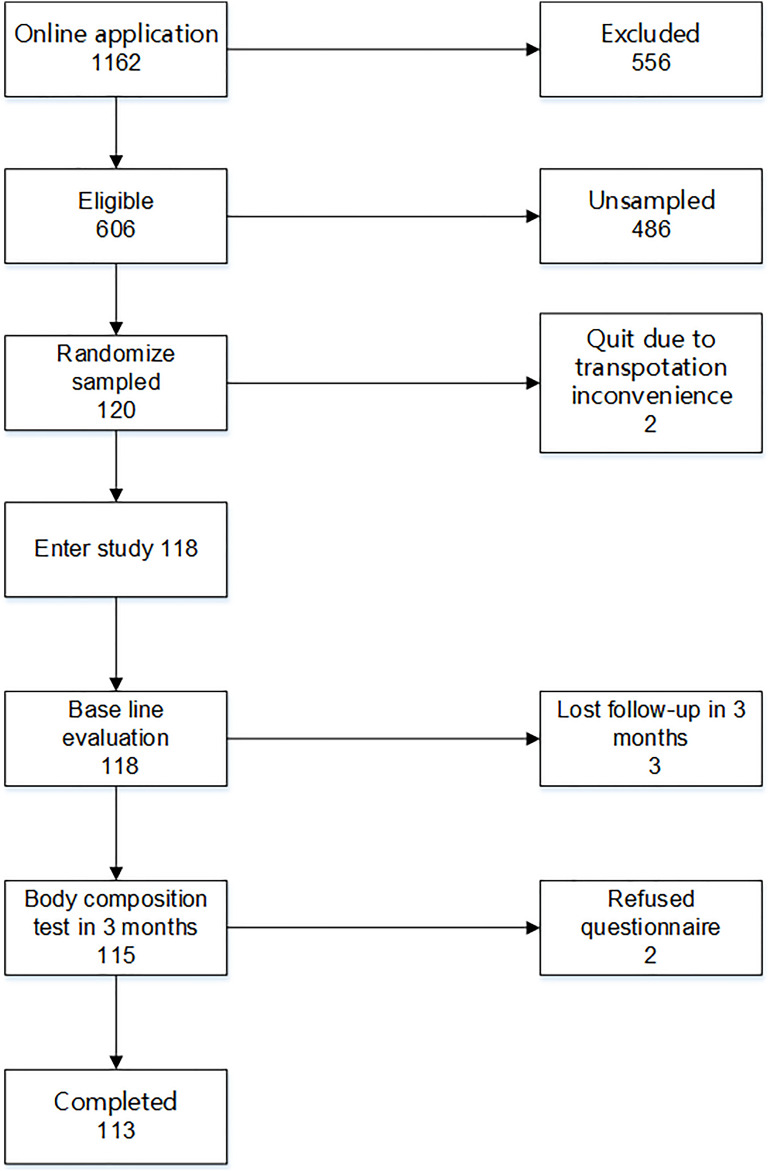
Flow diagram of participants in the program.

From November 8, 2017 to March 22, 2018, 118 eligible patients were randomly recruited in the study and completed the baseline assessment. Then, each participant was invited to an online Wechat group community. After a 3-month intervention, 113 patients completed the final assessment; two refused to take the 3-month assessment due to inconvenience in transportation, and three completed the body composition test in 3 months but refused to answer the questionnaire (Figure 1). The physical activity data of 96 participants were synchronized to the patients’ health management system. The data of the other 22 participants could not be synchronized due to account authority.

### Baseline Characteristics of the Participants

The average age of the participants was 48.83 ± 8.44 years, with the youngest being 27 years old and the oldest being 65 years old. The median time to surgery was 2.26 (1.55–3.64) years. Furthermore, 52.21% of the participants had college education and 92.4% were married. The proportion of patients with local and regional breast cancer was 61.06 and 38.94%, respectively, with 72.57% luminal type, 9.73% HER-2-enriched, and 15.93% triple-negative. The participants with a normal BMI (18.5–23.9 kg/m^2^) accounted for 65.49%. The body composition at baseline showed that 41.59% of patients had SMM lower than normal [skeleton muscle index: SMM (kg)/height (m)^2^ < 7.8 kg/m^2^] (23), and the PBF of 71.68% of patients was more than 30%, which was an average PBF in women aged 45–49 years (24) (Table 1).

**TABLE 1:** Baseline characteristics of the participants.

Characteristics	*n* = 113	Characteristics	*n* = 113
Age (mean ± SD) year	48.83 ± 8.44	Lymph node	
Time to surgery (year)		Negative	69 (61.06)
1∼	49 (43.36)	Positive	44 (38.94)
2∼	23 (20.35)	Chemotherapy	
3+	41 (36.28)	Yes	101 (89.38)
Education		No	12 (10.62)
Junior school	12 (10.62)	Endocrine therapy	
High school	34 (30.09)	Yes	82 (72.57)
College	59 (52.21)	No	31 (27.43)
Postgraduate	8 (7.08)	Molecular classification	
Marriage		Luminal A and B	82 (72.57)
Never married	3 (2.65)	HER-2 enriched	11 (9.73)
Married	104 (92.04)	Triple negative	18 (15.93)
Divorced/Separated	5 (4.42)	Not identified	2 (1.77)
Widowed	1 (0.88)	BMI (kg/m^2^)	
Employment		<18.5	6 (5.31)
Employed/Retired	87 (76.99)	18.5–23.9	74 (65.49)
Unemployed	8 (7.08)	24–27.9	26 (23.01)
Others	16 (14.16)	≥28	7 (6.19)
Household income per capita		SMM	
< ¥6000	35 (30.97)	Lower	47 (41.59)
¥6000–14,999	57 (50.44)	Normal	65 (57.52)
≥ ¥15,000	21 (18.58)	Higher	1 (0.88)
Menopausal status		PBF (%)	
Premenopausal	39 (34.51)	<30	32 (28.32)
Postmenopausal	74 (65.49)	≥30	81 (71.68)

### Physical Activity Monitoring

The median (quartile) wear days were 91 (89–91) days, with a minimum of 5 days due to lost equipment.

The number of daily walk steps of the participants was 8,851.28 ± 2,399.31, with median of 143.52 kcal physical activity consumption. The daily walking time was 95.97 ± 20.25 min on average, with 59.21% days reaching the set goal calorie consumption. The median running time was 5.11 min, with the first and the third quartiles being 2.77 and 8.28 min, respectively (Table 2).

**TABLE 2:** Physical activity status monitored using a wearable activity tracker.

Characteristics	Value
Daily step counts, mean ± SD	8851.28 ± 2399.31
Daily PA calories (kcal), median (quartiles)	143.52 (116.06–170.13)
Daily walking time (minutes), mean ± SD	95.97 ± 20.25
Daily running time (minutes), median (quartiles)	5.11 (2.77–8.28)
Accumulated days	8314
Reach standard days (%)	4923 (59.21%)

### Body Composition Change in 3 Months

After a 3-month intervention, significant changes were observed in body weight, fat mass, BMI, PBF, BMR, and VFA; VFA had the most significant changes (–6.54 cm^2^). No significant changes were observed in protein mass and SMM (Table 3).

**TABLE 3:** Body composition change after 3-month wearable activity tracker intervention.

Body composition	Baseline	In 3 months	Differences (95% CI)	*t*	*P* value
Body weight (kg)	57.19 ± 7.73	56.59 ± 7.70	–0.61 (–0.87 to –0.34)	4.555	<0.001*
Protein mass (kg)	7.43 ± 0.79	7.46 ± 0.78	0.03 (–0.01 to 0.07)	–1.322	0.189
Fat mass (kg)	19.19 ± 5.17	18.38 ± 5.16	–0.81(–1.07 to –0.55)	6.149	<0.001*
SMM (kg)	20.41 ± 2.39	20.52 ± 2.35	0.11 (–0.01 to 0.23)	–1.893	0.061
BMI (kg/m^2^)	22.77 ± 2.80	22.53 ± 2.79	–0.24 (–0.34 to –0.13)	4.576	< 0.001*
PBF (%)	33.10 ± 5.32	32.00 ± 5.46	–1.10 (–1.47 to –0.73)	5.883	< 0.001*
BMR(kJ/m^2^/h)	1190.86 ± 86.24	1195.22 ± 85.56	4.36 (0.26–8.46)	–2.106	0.037*
VFA (cm^2^)	91.01 ± 33.13	84.15 ± 32.09	–6.86 (–8.94 to –4.78)	6.539	< 0.001*

*Statistically significant at α = 0.05.

Body weight and BMI were reduced in patients who had chemotherapy, patients with or without endocrine therapy, patients with luminal A and luminal B, triple-negative patients, patients who had undergone surgery, patients with or without lymph node positivity, patients with BMI≥ or <24 kg/m^2^, and patients of any age. Protein mass and SMM increased in patients without endocrine therapy, HER-2-enriched patients, lymph node-negative patients, patients with BMI <24 kg/m^2^, and patients aged more than 45 years. Fat mass, PBF, and VFA decreased in patients with chemotherapy, patients with or without endocrine therapy, patients with luminal A and luminal B, HER-2-enriched patients, patients who had undergone surgery, and patients with any BMI category and age (Table 4).

**TABLE 4:** Body composition change in different subgroups.

Characteristics		Change mean (95% CI)							
	*N*	Body weight	Protein mass	Fat mass	SMM	BMI	PBF	BMR	VFA
**Adjuvant chemotherapy**									
Yes	101	–0.66* (–0.95 to –0.37)	0.03 (–0.01 to 0.08)	–0.88* (–1.16 to –0.60)	0.12 (–0.01 to 0.25)	–0.26* (–0.37 to –0.15)	–1.17* (–1.57 to –0.78)	4.64* (0.13–9.15)	–7.11* (–9.28 to –4.94)
No	12	–0.27 (–1.03 to 0.48)	–0.02 (–0.10 to 0.06)	–0.29 (–1.23 to 0.64)	0.04 (–0.19 to 0.27)	–0.10 (–0.40 to 0.20)	–0.56 (–1.87 to 0.75)	0.50 (–11.04 to 12.04)	–5.69 (–14.14 to 2.76)
**Endocrine therapy**									
Yes	82	−0.64* (–0.97,-0.30)	0.01 (–0.05 to 0.06)	–0.75* (–1.06 to –0.43)	0.06 (–0.09 to 0.20)	–0.24* (–0.37 to –0.11)	–0.97* (–1.41 to –0.52)	2.33 (–2.77 to 7.43)	–6.49* (–9.02 to –3.96)
No	31	–0.58* (–1.01 to –0.15)	0.08* (0.01–0.16)	–1.00* (–1.50 to –0.49)	0.24* (0.04–0.43)	–0.24* (–0.41 to –0.07)	–1.47* (–2.20 to –0.74)	9.06* (1.92–16.21)	–8.18* (–12.01 to –4.35)
**Molecular**									
Luminal A and luminal B	82	–0.65* (–0.99 to –0.31)	0.02 (–0.04 to 0.07)	–0.81* (–1.13 to –0.48)	0.09 (–0.06 to 0.23)	–0.25* (–0.38 to –0.12)	–1.08* (–1.54 to –0.61)	3.36 (–1.80 to 8.52)	–6.78* (–9.34 to –4.21)
HER-2 enriched	11	–0.37 (–1.08 to 0.33)	0.17* (0.04–0.30)	–1.15* (–1.71 to –0.58)	0.44* (0.08–0.79)	–0.16 (–0.43 to 0.10)	–1.85* (–2.71 to –0.98)	16.73* (3.41–30.05)	–11.03* (–16.24 to –5.81)
Triple negative	18	–0.67* (–1.24 to –0.11)	–0.02 (–0.10 to 0.06)	–0.64 (–1.33 to 0.05)	–0.02 (–0.26 to 0.22)	–0.27* (–0.50 to –0.04)	–0.73 (–1.67 to 0.21)	–0.72 (–9.03 to 7.59)	–5.78* (–11.29 to –0.28)
Not identified	2	–0.25 (–13.59 to 13.09)	0.10 (–1.17 to 1.37)	–0.90 (–21.23 to 19.43)	0.40 (–4.68 to 5.48)	–0.10 (–5.18 to 4.98)	–1.70 (–29.65 to 26.25)	13.50 (–132.62 to 159.62)	–2.55 (–95.94 to 90.84)
**Time to surgery**									
1∼	44	–0.64* (–1.01 to –0.26)	0.05 (–0.01 to 0.12)	–0.97* (–1.39 to –0.56)	0.21* (0.04–0.37)	–0.25* (–0.40 to –0.10)	–1.39* (–1.98 to –0.79)	7.31* (1.48–13.14)	–8.87* (–12.42 to –5.32)
2∼	101	–0.67* (–1.17 to –0.17)	0.01 (–0.08 to 0.11)	–0.72* (–1.34 to –0.10)	0.00 (–0.29 to 0.29)	–0.26* (–0.46 to –0.06)	–0.93* (–1.80 to –0.06)	1.13 (–8.41 to 10.68)	–5.34* (–10.39 to –0.29)
3+	12	–0.57* (–1.11 to –0.03)	0.00 (–0.07 to 0.08)	–0.69* (–1.12 to –0.25)	0.06 (–0.16–0.27)	–0.22* (–0.43 to –0.01)	–0.88* (–1.50 to –0.26)	2.27 (–5.53 to 10.07)	–5.62* (–8.56 to –2.69)
**Lymph node**									
Negative	69	–0.42* (–0.71 to –0.12)	0.05* (0.00–0.10)	–0.68* (–1.01 to –0.35)	0.15* (0.02–0.29)	–0.16* (–0.28 to –0.04)	–1.00* (–1.48 to –0.53)	5.57* (0.60–10.53)	–6.28* (–8.90 to –3.65)
Positive	44	–0.95* (–1.46 to –0.44)	–0.01 (–0.09 to 0.07)	–1.03* (–1.49 to –0.58)	0.04 (–0.18 to 0.26)	–0.37* (–0.57 to –0.18)	–1.28* (–1.92 to –0.64)	2.00 (–5.58 to 9.58)	–8.05* (–11.60 to –4.50)
**Baseline BMI (kg/m^2^)**									
≥24	35	–0.98* (–1.64 to –0.33)	–0.03 (–0.12 to 0.06)	–0.90* (–1.42,-0.37)	–0.09 (–0.33 to 0.15)	–0.38* (–0.63 to –0.14)	–0.87* (–1.43 to –0.32)	–1.91 (–10.34 to 6.53)	–7.22* (–11.26 to –3.18)
<4	83	–0.45* (–0.71 to –0.19)	0.05* (0.01–0.10)	–0.77* (-1.08,-0.47)	0.19* (0.06–0.32)	–0.18* (–0.28 to –0.07)	–1.19* (–1.67 to –0.72)	6.91* (2.27–11.56)	–6.71* (–9.19 to –4.24)
**Age group**									
≤45	42	–0.76* (–1.31 to –0.22)	–0.03 (–0.11 to 0.06)	−0.70* (–1.17,-0.22)	–0.07 (–0.29 to 0.16)	–0.29* (–0.50 to –0.08)	–0.85* (–1.54 to –0.16)	–1.31 (–9.15 to 6.53)	–4.67* (–7.98 to –1.37)
>45	71	−0.53* (–0.82to –0.25)	0.06* (0.01–0.11)	–0.89* (–1.21 to –0.57)	0.22* (0.09–0.35)	–0.21* (–0.33 to –0.10)	–1.26* (–1.71 to –0.82)	7.50* (2.79–12.21)	–8.33* (–11.03 to –5.63)

*Statistically significant at α = 0.05.

### Adverse Effects

After the intervention, 99% participants were satisfied with the program, and 110/113 were willing to continue wearing the device. Two had a dermatological allergy, three had a psychological burden (worried about not completing the calorie consumption goal), and one had uneasiness in sleeping as adverse effects.

## Discussion

This study showed that the body composition of breast cancer survivors improved through a wearable technology-based 3-month intervention. Body weight, BMI, fat mass, PBF, BMR, and VFA also significantly decreased. The effect was consistent within most strata, indicating that patients could benefit from the intervention irrespective of disease characteristics and treatment. Loss of body fat mass was most significant in luminal A and luminal B subgroups in which patients were more sensitive to estrogen and aromatase released during adipocyte expansion.

Although 65.49% of survivors showed a normal BMI at baseline assessment in this randomly selected sample, 71.68% of survivors had a PBF more than 30%, and 41.59% had low SMM, indicating a poor body composition among breast cancer survivors. Obesity is related to poorer outcomes with increased mortality among survivors (6, 25, 26), and most patients with breast cancer would have a weight gain after diagnosis (27).

A recent study showed that wearable technology-based intervention effectively increased the MVPA and reduced the sedation time (28). Studies also showed that wearable devices increased self-awareness and motivation for physical activities. Doctor monitoring and peer support were also important in the use of wearable activity trackers (29). Although the daily step counting was more than 8,800, the running time was very low among the participants, with a median time of only 5.11 min daily. The physical activity pattern varied from person to person. Studies suggested that patients planning to lose weight could better benefit from a continuous aerobic exercise in which the heart rate reached the target heart rate or caused energy deficit, as in the LISA (30) and ENERGY (31) study. However, the present study showed that, even if the exercise intensity was far from vigorous with enough time of moderate exercise, breast cancer survivors could still obtain benefits from the improvement in body weight and body composition. However, the improvement was mainly in terms of a decrease in body fat, while protein mass and SMM showed no significant improvement. This could be due to intervention monitoring and setting the goal for calorie consumption, with no track of anti-resistance training, which improved the SMM. It was also a safer way, compared with high-intensity exercise, for breast cancer survivors whose average age was around 50 years since a large number of hormone receptor-positive patients received aromatase inhibitor drugs for a long time, causing osteoporosis (32). A recent study also showed that older women with an average of 7,500 steps/day had reduced all-cause mortality, and the benefit was not associated with intensity (33).

Since breast cancer has a high incidence rate and a relatively good prognosis, the number of breast cancer survivors is huge. The 5-year prevalence of breast cancer is nearly 1.1 million in China alone (34). As an appropriate technology to be generalized, the wearable technology was considered to be one of the choices with preferable features of safety, acceptability, and cost-effectiveness. Besides causing an improvement in body composition, this low-cost comprehensive care, including body composition analysis, health education, and wearable device application, is welcomed by participants with nearly no harm, strong interaction, and fun. This study used a wearable activity tracker to monitor the activity, which could reflect the objective and the real status of each participant. Besides BMI, body composition might be an additional indicator to monitor the achievement of lifestyle intervention. In addition to breast cancer, colorectal cancer, esophageal adenocarcinoma, and endometrial cancer share a common risk factor of body fat. The wearable technology also has the potential to be applied in the long-term survival management of patients with postoperative early stage cancer. Its benefits need further investigation.

However, the present study also had some limitations. First, the baseline physical activity assessment before the wearable equipment was delivered was hard to collect, making it difficult to compare the physical activity volume and contributing to the effects of the increase in physical activity volume. Attempts were made to evaluate the activity status before the intervention, but the data were far from consistent with what the wearable equipment collected. Second, the study was not designed as a randomized controlled trial because the technology was easy to be accessed by those not even enrolled in the study and the patients in the center had frequent interaction and communication, which might have led to contamination of the randomized controlled trial. Hence, it was hard to identify which part of the interventions contributed to the benefit during the whole intervention period. Third, the result of this study could not be generalized to all patients with breast cancer because patients in the advanced stage were not included. The management of advanced-stage patients would be totally different due to different treatment regimens and physical conditions, especially patients with cachexia. Fourth, the participants were all patients capable of using wearable devices, which compromised the representativeness of the patients. When upscaling the intervention, such factors should be taken into consideration, and an improvement in usability needs to be further optimized.

Future studies should involve more indicators, especially biomarkers such as leptins, C-reactive protein, and TNF-α, and should have expanded sample size and better study design to better reflect the effect of wearable technology intervention on breast cancer survivors.

## Conclusion

The wearable technology-based comprehensive intervention can help breast cancer survivors in controlling body weight and improving body composition, especially in reducing body fat, thus improving the prognosis. The effect was consistent within most strata in this study, indicating that patients with different age, staging, and molecular profile and with or without chemotherapy or endocrine therapy could benefit from the intervention.

## Data Availability Statement

The raw data supporting the conclusions of this article will be made available by the authors, without undue reservation.

## Ethics Statement

The studies involving human participants were reviewed and approved by Fudan University Shanghai Cancer Center Research Committee. The patients/participants provided their written informed consent to participate in this study.

## Author Contributions

CZ and YZ conceived and designed the project and wrote the manuscript. ZS and YZ supervised the study. JC, LT, and JQ recruited the participants. MM, CZ, ZW, and JS collected the data. YL and HD provided body composition test and nutritional consulting for the participants. QJ provided a patient’s management platform. HW provided the data interface transferring wearable equipment data to the database. All the authors contributed to the study conception and design, read, reviewed, and approved the final manuscript.

## Conflict of Interest

HW was employed by Huami Information Technology Co., Ltd. and QJ was employed by Shanghai Ruochu Information Technology Co., Ltd. The remaining authors declare that the research was conducted in the absence of any commercial or financial relationships that could be construed as a potential conflict of interest.
